# Tactical treatment with copper oxide wire particles and symptomatic levamisole treatment using the FAMACHA^©^ system in indigenous goats in South Africa

**DOI:** 10.1016/j.vetpar.2011.08.003

**Published:** 2012-02-28

**Authors:** A. Spickett, J.F. de Villiers, J. Boomker, J.B. Githiori, G.F. Medley, M.O. Stenson, P.J. Waller, F.J. Calitz, A.F. Vatta

**Affiliations:** a Onderstepoort Veterinary Institute, Private Bag X05, Onderstepoort 0110, South Africa; b KwaZulu-Natal Department of Agriculture, Environmental Affairs and Rural Development, Private Bag X6005, Hilton 3245, South Africa; c University of Pretoria, Private Bag X04, Onderstepoort 0110, South Africa; d International Livestock Research Institute (ILRI), P.O. Box 30709, Nairobi 00100, Kenya; e University of Warwick, School of Life Sciences, Coventry CV4 7AL, United Kingdom; f National Veterinary Institute (SVA), Department of Parasitology (SWEPAR), Uppsala SE-751 89, Sweden; g ARC-Biometry Unit, 1134 Park Street, Hatfield 0083, South Africa

**Keywords:** Copper oxide wire particles, FAMACHA^©^, *Haemonchus contortus*, Indigenous goats, Levamisole, Tactical treatment

## Abstract

Haemonchosis is considered to be the most economically important gastrointestinal disease of small ruminants in the tropics and subtropics. However, chemical anthelmintics, which were the mainstay of control, have been compromised by a high prevalence of resistance worldwide. Copper oxide wire particles (COWP) have been shown to have anthelmintic effects, but few studies have examined their use under field conditions. The use of COWP was therefore evaluated as a tactical anthelmintic treatment in indigenous goats raised under communal farming conditions in Bergville, KwaZulu-Natal Province, South Africa. At the beginning of the summer rainfall season (October 2007), the faecal egg counts of 172 female goats belonging to 15 farmers were determined and this sampling continued every four weeks until the second week of January 2008. The goats within each of the 15 herds were ranked according to their faecal egg counts for this week. The goats were sequentially paired off within each ranking starting with those goats with the highest counts. One goat from each pair was randomly allocated to a treated or control group. Two weeks later, a 4 g COWP bolus was randomly administered to each goat in the treated group. Faecal egg counts were carried out on the goats two weeks following treatment, and the sampling of the goats then proceeded every four weeks until October 2008. Except for the six-week period prior to the administration of the COWP, the goats were examined according to the FAMACHA^©^ system and symptomatically treated with 12 mg/kg levamisole when anaemic. The percentage reduction in faecal egg count due to the COWP treatment was 89.0%. Mean pre- and post-treatment faecal egg counts for the COWP-treated group (*n* = 73) were 2347 eggs per gram of faeces (epg) and 264 epg, respectively. The corresponding values for the untreated controls (*n* = 66) were 2652 epg and 2709 epg. The prevalence of *Haemonchus* spp. larvae in pre- and post-treatment faecal cultures was 72% and 46%, respectively. Symptomatic anthelmintic treatments in combination with mid-summer tactical treatments with COWP appear to be useful strategies for the control of *Haemonchus contortus* in indigenous goats in this farming system and this approach could have application in other similar agro-ecological zones.

## 1 Introduction

Haemonchosis, caused by the abomasal nematode, *Haemonchus contortus*, is a common and severe disease of small ruminants in the tropics and subtropics. It is ranked amongst the most important diseases of small ruminants in the summer rainfall area of South Africa (Vatta et al., 2001). The use of chemical anthelmintics was the main method to control the disease. Approximately US$ 17.6 million was spent in South Africa alone during the year 2003/2004 on anthelmintics for sheep and cattle and endectocides for all animal species (Vatta and Lindberg, 2006). However, control of *H. contortus* has been complicated by anthelmintic resistance, which has become a global problem. This is of particular concern in countries where the sheep and goat industries are well developed (Love et al., 2003), but even within the small-scale farming sectors of developing countries, resistant worm populations have been reported (Vatta and Lindberg, 2006). Consequently, a variety of alternatives for worm management are being researched, including the use of copper oxide wire particles or COWP (Bang et al., 1990b; Burke et al., 2004; Knox, 2002; Martínez Ortiz De Montellano et al., 2007).

COWP technology offers considerable promise as a means of control in the *H. contortus* endemic regions of Africa, but needs to be comprehensively assessed in individual breeds and within different farming systems. One aims of this study was to examine the efficacy of COWP as a tactical anthelmintic treatment in indigenous goats raised under communal grazing conditions in South Africa. A second aim was to examine the usefulness of the tactical COWP treatment when used in combination with monthly symptomatic treatments of the goats with levamisole by means of the FAMACHA^©^ system (Malan et al., 2001; Vatta et al., 2001).

## 2 Materials and methods

### 2.1 Study site and identification of participating farmers

**Fig. 1:**
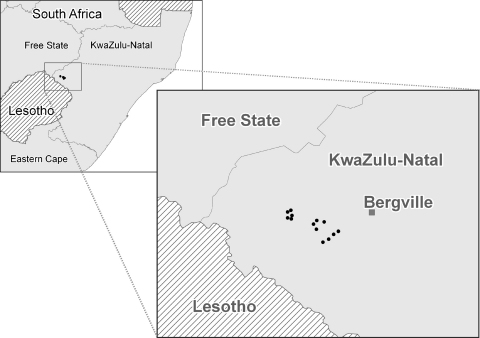
Study area: localities of homesteads near Bergville in KwaZulu-Natal Province, with the adjacent provinces of the Free State and Eastern Cape shown.

The study was conducted as an on-farm experiment utilizing goats belonging to small-scale farmers in the Bergville area in KwaZulu-Natal Province, South Africa. Prior participatory appraisal exercises with the community had identified haemonchosis as a problem in the goats and fifteen participating farmers from three areas, Hoffenthal, Ogade and Dukuza (Fig. 1), were selected to participate in an on-farm research project. Farmers owned between 14 and 150 goats which were grazed on communal land during the day and housed in simple facilities at night. Each farmer participating in the experiment undertook to reserve at least eight female goats for the experiment as female goats were not likely to be sold or slaughtered as readily as males. Site localities and altitudes were determined using a GARMIN eTrex^®^ Vista Cx Global Positioning System (GPS) device (Garmin Distribution Africa (Pty) Ltd.).

### 2.2 Climate and vegetation of the study area

The vegetation and the climatic conditions of the study area falls within Biosource Groups 11 (Moist Transitional Tall Grassveld) and 12 (Moist Tall Grassveld) (Camp, 1999), which for the most part is found at altitudes ranging from 900 to 1400 m and is characterized by the abundance of thatch grass, *Hyparrhenia hirta*. The average mean annual rainfall ranges from 712 to 1116 mm per annum. The rainy season generally occurs from September (spring) to May with rainfall increasing to December (mid-summer), and then declining towards May (late autumn). Pastures are infective for *H. contortus* from about November onwards. Temperatures range from 12 to 29 °C (mean 20.5 °C) in summer and 1 to 23 °C (mean 12 °C) in winter.

Data for rainfall and temperature during the study period were obtained from the weather station, Broad Acres, Bergville (28°49′7.14″S; 29°24′7.74″E; altitude 1246 m) which became operational in 2003.

### 2.3 Epidemiological investigations

The field experiment was conducted from October 2007 until November 2008 and a total of 189 animals were included in the experiment. The animals used are referred to in this article as “indigenous”, as they had the general appearance of the goats traditionally kept by the IsiZulu-speaking people of the area for sale and traditional slaughter. There appeared to some Boer goat influence in the herds. None of the herds could be classified as belonging to a distinctly different breed from any of the other herds. The faecal egg counts (FECs), packed cell volumes (PCVs), body condition scores (BCSs) and live weights of the goats were monitored every four weeks starting during the week of 15 October 2007 and continuing until the week of 8 January 2008. During the week of 22 January 2008, COWP boluses were administered orally to the treated group, as described below. The animals were sampled two weeks later, in the week of 5 February 2008, and the monitoring continued every four weeks after that until and including the week of 13 October 2008. The goats were aged by examining the incisor teeth (Mitchell, 1982), assigned unique identification numbers and individually ear-tagged. Young animals (0 permanent incisor teeth) and adults (≥2 permanent incisor teeth) were identified as such at the commencement of the trial (October 2007). The samples were collected over a three day period in each week of sampling. The area visited and the individual farms were consistently visited on the same day of the week (Tuesday, Wednesday or Thursday) during each field trip.

Faecal egg counts in eggs per gram of faeces (epg) were carried out using the Pitchford–Visser method (Reinecke, 1983), as modified by Malan and Visser (1993). Excess faeces not used for the faecal egg counts were pooled for each herd and cultured for infective third-stage larvae (Van Wyk et al., 2004). Differentiation between *Teladorsagia* and *Trichostrongylus* spp. was not attempted. Faeces were collected from additional goats not included in the experiment in each herd and added to the pooled faecal sample before culture. One hundred larvae harvested from each culture were identified except where the culture did not yield 100 larvae. In such cases, as many larvae as possible were identified. The body condition of each goat was scored on a scale of 1 (very thin)–5 (very fat) (Russell, 1984; Williams, 1990). The live-weights were determined using a spring balance (Salter Model 235, Capital Scales, Pretoria, South Africa) suspended from a tripod.

All the goats belonging to each farmer were monitored for FAMACHA^©^ score. Using this system, each goat is assigned a score of 1 (non-anaemic)–5 (severely anaemic) based on the colour of the conjunctival mucous membranes. All the experimental animals that had a FAMACHA^©^ score of 3, 4 or 5 were treated with 12 mg/kg levamisole (Tramisol, Coopers, Afrivet, South Africa). A faecal egg count reduction test conducted on goats in the study area at the start of this study indicated that levamisole had an efficacy of 98.5% at a dosage of 12 mg/kg. Packed cell volumes for each animal were determined according to the microhaematocrit method (Vatta et al., 2007) on the same day as the FAMACHA^©^ scores were assigned. When it was found that an animal had a PCV of less than 20% but had not been assigned a FAMACHA^©^ score of 3, 4 or 5 and hence had not been treated with anthelmintic on the day of examination, such animal was then subsequently treated with 12 mg/kg levamisole before the end of the week in question.

The scoring and appropriate treatment of the animals according to the FAMACHA^©^ system took place at each visit except for the weeks of 11 December 2007, 8 January 2008 and 22 January 2008 (the date the COWP was administered), when the goats were scored but not actually treated. The 6 week period that was free from treatment was to raise pasture contamination in order to ensure a high nematode challenge.

### 2.4 Testing of copper oxide wire particles

The experimental goats of each of the 15 participating farmers were allocated to a COWP-treated and an untreated control group. This was done by ranking the goats for each farmer from highest to lowest according to their faecal egg count for the sampling week of 8 January 2008. The goats were then sequentially paired starting with those with the highest egg counts and one of the two were randomly assigned to the treated or the control group. The remaining one of the pair was then allocated to the group not allocated to the first of the pair.

During the sampling week of 22 January 2008, 4 g COWP boluses (Copinox^®^, Animax Veterinary Technology, United Kingdom) were administered orally with an applicator, to those animals that had been allocated to the COWP treated group. Directly after the bolus had been given, 10 ml of water was administered to the animal using a syringe. The animals were carefully observed to ensure that the bolus had been swallowed. Animals assigned to the control group received 10 ml of water only administered by syringe *per os.* The faecal egg counts for the sampling week of 22 January 2008 (pre-COWP) and the sampling week of 5 February 2008 (2 weeks post-COWP) were used to calculate the percentage reduction in egg counts (percentage efficacy) as a result of the COWP administration. Goats for which egg counts were not present at both sampling occasions as well as those with egg counts of less than 200 epg pre-COWP-treatment were excluded from the calculations. An animal whose PCV was below 20% during the sampling week of 8 January 2008 and was treated with levamisole, was also excluded from the calculations. COWP efficacy was calculated using the formula by Presidente (1985), but using the arithmetic means:

$$\text{Percentage}\;\text{reduction}\;(\%\;\text{efficacy})=\left[1-\left(\frac{\text{PoT}}{\text{PrT}}\right)*\left(\frac{\text{PrC}}{\text{PoC}}\right)\right]*100$$

PoT = post-treatment mean faecal egg count of the treated group; PrT = pre-treatment mean faecal egg count of the treated group; PrC = pre-treatment mean faecal egg count of the control group; PoC = post-treatment mean faecal egg count of the control group.

### 2.5 Data analysis

Seventeen goats were absent at two or more sampling occasions and all the data for these goats were excluded from the analysis of the epidemiological investigations. A total of 172 goats were included in these final analyses, 55 from the Hoffenthal area (5 farmers), 45 from the Ogade area (6 farmers) and 72 from the Dukuza area (4 farmers). The data were entered into an MS-Excel spreadsheet and an analysis of variance (ANOVA) (using Genstat^®^ for Windows^®^ 13th edition, VSN International Ltd.) was performed to test for differences between the control and COWP-treated groups of goats in their mean FECs, FAMACHA^©^ scores, PCVs and BCSs. Faecal egg counts were transformed (log_10_) to stabilize variances for the analyses but the untransformed means were used in the figures in the present paper. Testing was done at the 5% confidence level and the means separated using Fisher's Protected Least Significant Difference (LSD) test.

The FEC and PCV data were further subjected to Levene's test (Levene, 1960) for homogeneity of variances between sites for four separate times with regard to COWP administration: pre-COWP, and 2, 6 and 10 weeks post-COWP. Thereafter the combined data were analyzed as a three-factor factorial in a completely randomized design at the four separate times. The factors were the three areas (Hoffenthal, Ogade and Dukuza), two treatments (control and COWP) and two ages (young and adult). The appropriate analysis of variance (ANOVA) was performed (using Genstat^®^ for Windows^®^ 13th edition, VSN International Ltd.) on the transformed (log_10_) FECs and the PCVs. Again, Fisher's Protected Least Significant Difference was calculated at a 5% significance level to compare means of significant effects.

A Spearman's rank correlation coefficient was calculated to examine the relationship between the FAMACHA^©^ scores and PCV values obtained.

## 3 Results

### 3.1 Climatological data

**Fig. 2:**
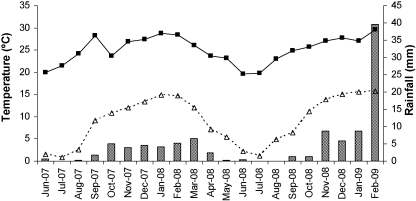
Mean average rainfall (columns) and minimum (▵) and maximum (■) temperatures in the Bergville area throughout the trial period.

Mean average rainfall and minimum and maximum temperatures recorded throughout the study period are given in Fig. 2.

Seasonal rainfall occurred from September 2007 to April 2008, tapered off during May to August 2008 and started again in September 2008. Maximum temperatures were seen in January 2008 and February 2009. Winter minimum temperatures occurred in July.

### 3.2 Live weights and body condition score

The young goats had an average live weight ± standard deviation of 24.75 ± 2.89 kg while the adult goats weighed 33.61 ± 2.03 kg. Live-weight data were used to determine the drug dose at the time of sampling but were not further analyzed because of the effects of pregnancy and kidding on live weight. The mean BCSs for the goats fluctuated minimally over the trial period with no significant differences between the control and treated groups of goats being evident. The BCSs of the adults ranged between 1.73 and 2.41 for the controls and 1.85 and 2.45 for the treated group of goats.

### 3.3 Faecal egg counts

**Fig. 3:**
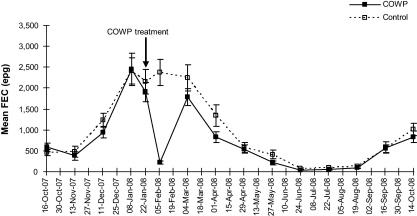
Mean faecal egg counts (FECs) for the control and COWP-treated goats combined for all three trial areas. The error bars indicate the standard error.

An increase in the mean FEC was recorded from November 2007 to January 2008 (Fig. 3) and the egg counts in the controls remained relatively high (>2000 epg) into March 2008. There was a steady seasonal decline in mean FEC from April to May 2008, the FECs reaching negligible numbers during the winter month of June 2008. Mean FECs started to increase again in September 2008.

The analysis of variance (ANOVA) for pre- and three periods post-COWP administration for log_10_ faecal egg counts and PCV values are given in Tables 1a and 1b. No significant evidence of homogeneity of variances were found between sites for the transformed FECs except for two weeks post COWP (*P* = 0.025; Table 1a). All the other periods had a *P* > 0.576. As such, the site variances could be considered of comparable magnitude and a valid factorial ANOVA was done on the data combined for the three sites.

The ANOVAs indicated no significant two or three factor interactions, therefore only the main effects were examined. Tables 2a and 2b gives the main effect means for the transformed and untransformed FECs and PCV values.

During the week of 5 February 2008, two weeks after COWP administration (week of 22 January 2008), a marked decrease in FEC for the COWP-treated group of goats was evident. There was strong evidence (*P* < 0.001) of a difference in FEC between the controls (2382 epg) and the treated group (223 epg) (see Fig. 3 and Table 2a). The egg counts in the treated group had increased by the next sampling event (week of 4 March 2008), six weeks after COWP administration and no difference in egg counts was evident between the treated and untreated groups on this date (*P* = 0.787), nor was any difference noted at ten weeks post-COWP (*P* = 0.256; Table 2a).

The FECs for the goats in Dukuza were higher than those for Hoffenthal and Ogade at two weeks post-COWP (*P* = 0.004) and higher than those for Ogade at ten weeks post-COWP (*P* = 0.016). The FECs for the young goats were higher than those for the adults at 6 and 10 weeks post-COWP (*P* = 0.008).

The overall egg count reduction efficiency was 89.0%. The greatest COWP efficacy was in the Ogade area (95.8%), followed by Hoffenthal (91.2%) and Dukuza (81.8%). The FEC reduction was 85.5% in the young animals and 89.4% in the adults (Table 3).

### 3.4 Faecal cultures

**Fig. 4:**
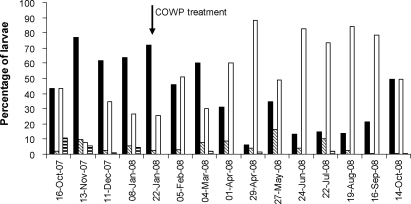
Percentage of nematode larvae recovered from faecal cultures, averaged for all three trial areas, identified as *Haemonchus* (solid black columns), *Oesophagostomum* (diagonally striped columns), *Teladorsagia/Trichostrongylus* (white columns) and *Strongyloides* (horizontally striped columns) for each sampling occasion of the trial period.

Four genera were identified in faecal culture, i.e. *Haemonchus, Oesophagostomum, Teladorsagia/Trichostrongylus* and *Strongyloides*. Similar percentages of each genus were present at the three study sites. These were therefore averaged and are presented for each sampling week in Fig. 4. *Haemonchus* larvae were predominant during the rainy summer season from November 2007 to March 2008 with *Teladorsagia/Trichostrongylus* spp. second in abundance during this period. From April 2008 to October 2008, during the drier, autumn and winter months, this predominance was reversed. *Oesophagostomum* and *Strongyloides* larvae were present in low numbers throughout the trial period at most sampling occasions.

The COWP boluses were administered when peak faecal egg counts were recorded in January 2008, corresponding to peak *Haemonchus* spp. larvae numbers (71.9% of total larvae) which were almost 3-fold higher than those of the second most abundant group (*Teladorsagia/Trichostrongylus* spp.; 25.7% of total larvae). Although faecal cultures were not set up separately for the COWP-treated and control goats, an overall reduction in the percentage of *Haemonchus* spp. larvae (46.1% of total larvae) was seen two weeks after COWP was administered, while *Teladorsagia/Trichostrongylus* spp. proportions had doubled (50.9% of total larvae).

### 3.5 Packed cell volume

**Fig. 5:**
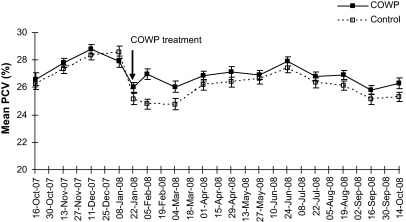
Mean PCV for the control and COWP-treated goats combined for all three trial areas. The error bars indicate the standard error.

Over×all, the mean PCV was above 26% at the start of the trial and this increased to greater than 28% in mid-December 2008 (Fig. 5). By the time of COWP administration, the mean PCV had dropped below 26%. Unlike the COWP treated group (average 26.05%), the PCVs in the control goats continued to decrease slightly into March 2008 (average 24.76%) after which they remained around the 26% mark for the remainder of the trial.

As for the transformed FECs, no significant evidence of homogeneity of variances were found between sites for PCV (*P* = 0.389; Table 1b) and a factorial ANOVA was therefore done on the data combined for the three sites. The factorial ANOVA indicated no significant two or three factor interactions, therefore only the main effects were examined and these are given in Table 2b.

There was strong evidence (*P* < 0.001) of a difference between the control (PCV = 24.8%) and the treated group (PCV = 26.7%) two weeks after COWP administration (week of 5 February 2008; Table 2b). A lower PCV (*P* = 0.028) for the control group extended to the week of 4 March 2008. The PCVs for the goats in Dukuza and Ogade were lower than those for Hoffenthal at two weeks post-COWP (*P* < 0.001).

### 3.6 FAMACHA^©^ score

For the goats in all three areas combined, the mean FAMACHA^©^ score ranged from 2.12 to 3.10 over the trial period (data not shown). The mean scores fluctuated minimally during the trial period and displayed very little difference between the control and treated groups of goats. The number of goats that required drenching at the start of the trial (in October 2007) in the COWP-treated and control groups decreased, respectively from 68 to 31 and 66 to 21 by the end of the trial period (in October 2008; Table 4). Before the administration of COWP, at the second January 2008 sampling, 46 of the COWP-treated goats and 41 of the controls would have required drenching (but were not actually treated, as discussed in Section 2). Two weeks post-COWP administration (in February 2008), 38 goats in the COWP-treated group and 42 in the control group required drenching. However, six weeks after COWP was given (in March 2008), the number of COWP-treated goats requiring drenching increased to 48 while those in the control group remained at 42.

**Fig. 6:**
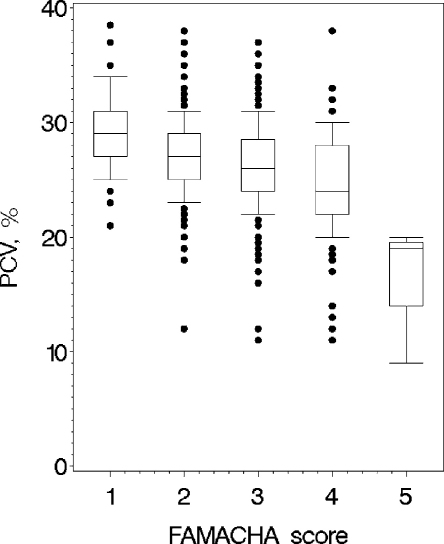
Box plots showing the relationship between PCV value and FAMACHA^©^ score category for all animals assessed during the trial. High and low bounds are at the 90th and 10th percentiles. Dots represent individual values outside of this range. The line inside the box indicates the median.

The Spearman's rank correlation coefficient between FAMACHA^©^ category scores and PCV values was −0.014 (−0.148 adjusted for ties), based on a sample size of 2563 (t approximation −7.56 on 2561 d.f., *P* < 0.001). Box plots demonstrating relationships between PCV values and FAMACHA^©^ score category are shown in Fig. 6.

## 4 Discussion

The present study provides basic epidemiological information on the gastro-intestinal nematode parasites in the indigenous goats raised by small-scale farmers under communal grazing conditions in the Bergville area, KwaZulu-Natal Province, South Africa. High temperatures and adequate rainfall during October, November and December contributed to good conditions on pasture for nematode larval survival. This was reflected in the high FECs seen in January, February and March (Fig. 3) and a corresponding decrease in the mean PCVs during this period (Fig. 5). The occurrence of a late warm wet season peak in FECs in the present study and a corresponding decrease in PCVs as a result of *H. contortus* infection agrees with the findings of Vatta et al. (2002, 2007).

Although differences in FECs and PCVs were evident between the areas following COWP administration, no consistent pattern was noted. This was also the case for differences in the FECs between the young and adult goats. However, a discernable effect of the COWP (which was given on 22 January 2008 during the period of higher egg counts) was seen two weeks after COWP administration when FECs in the treated groups of goats were reduced by 89.0%. This was mirrored by a corresponding increase in the relative mean PCV values (Fig. 5). At six weeks post-COWP administration (4 March 2008), the mean FEC of the treated group had increased to a level similar to that of the control group, whereas the effect on PCV extended for a six week period. Analysis of the faecal cultures suggests that the effect of the COWP was mainly, if not totally, on *Haemonchus* spp. The reduction in FECs following COWP administration was accompanied by a reduction in the percentage of *Haemonchus* spp. larvae in the cultures after treatment (Fig. 4). The reduction in the percentage of *Haemonchus* spp. larvae following COWP treatment may have been higher than the present results indicate, as COWP has only been shown to have an effect on abomasal parasites (Bang et al., 1990a). However, because the post-treatment cultures were not prepared separately for the treated and the untreated goats, the percentage reduction in *Haemonchus* spp. in the treated goats was probably masked to some extent by the presence of the nematode in the faeces of the control goats.

In several experiments in grazing goats, Burke et al. (2007b) also demonstrated a decrease in FECs after COWP administration, an effect which lasted 2–4 weeks. The effect of the COWP on FECs is substantiated by treatment and slaughter studies in sheep and goats. Burke et al. (2004) administered 2 g, 4 g and 6 g COWP to lambs and obtained reductions of 90%, 94% and 93% in four-week-old *H. contortus* counts, respectively. Using a 4 g COWP bolus in sheep, Waller et al. (2004) obtained 97% and 56% reductions in six week old burdens of adult and fourth stage larvae of *H. contortus*, respectively. Chartier et al. (2000) administered COWP at 2–4 g doses to dairy goats and obtained a 75% reduction in four-week-old *H. contortus* infections. In pen trials conducted as a precursor to this study, Vatta et al. (2009) obtained 95% and 93% reductions in six-week-old mean *H. contortus* counts in indigenous goats treated with 2 g and 4 g COWP, respectively. Soli et al. (2010) showed reductions of 67.2% and 85.8% in numbers of adult *H. contortus* in grazing sheep and goats, respectively. The animals were removed from pasture and slaughtered 28 days after treatment.

While the COWP boluses have an immediate anthelmintic effect, their extended effect is bounded. Vatta et al. (2009) also examined the extended effect of COWP in goats given 2–4 g COWP followed by a trickle infection of *H. contortus* larvae for six weeks. The COWP was ineffective in reducing the worm burdens in the treated versus the control goats in this developing infection group. The authors discussed that soluble copper levels in the abomasum were probably raised sufficiently high to kill abomasal nematodes for at most two weeks after COWP administration.

Because the administration of COWP to sheep and goats is associated with the risk of copper toxicity, Burke and Miller (2006) examined the administration of 0.5 g and 1 g COWP given at 6-week intervals for a total of four treatments in lambs. The COWP showed effective anthelmintic efficacy and the concentrations of copper in the liver at slaughter 28 days after the last treatment were normal. In addition, there appears to be less chance of copper toxicity due to the use of COWP in goats than in sheep. Vatta et al. (2009) found that tissue copper levels in goats treated with 4 g COWP did not differ significantly from those that were not treated 4 weeks after treatment. Further work under South African field conditions could examine the usefulness of additional follow-up treatments with COWP at lower doses than tested in the present study.

Vatta et al. (2007) examined appropriate strategies for nematode control, including the use of symptomatic and tactical anthelmintic treatment. In their study, these authors referred to symptomatic treatment as the anthelmintic treatment of goats considered to be anaemic according to the FAMACHA^©^ system. The goats were examined for anaemia at four-weekly intervals. Tactical anthelmintic treatment was defined as deworming when large worm burdens were expected (Van Schalkwyk et al., 1995). In the study by Vatta et al. (2007), a tactical ivermectin treatment was given in the mid-summer period to prevent the expected peak in faecal egg counts in the late summer. The present study suggests that COWP may be used as a potential effective alternative to the ivermectin used in that study.

In the present trial, it appears that the general decrease over the trial period in the number of goats requiring symptomatic anthelmintic treatment, as determined using the FAMACHA^©^ system, was similar for both the COWP-treated and control groups of goats and that the administration of COWP had an immediate but overall minimal effect in reducing the number of animals requiring treatment. The consistent use of the FAMACHA^©^ system in assigning drench applications over the trial period is probably the main reason for the decrease in drenching required at the end of the trial (Table 4).

As has been shown in other studies (Vatta et al., 2001; Kaplan et al., 2004; Burke et al., 2007a), the present study indicates a relatively wide overlap in PCV values for each FAMACHA^©^ score in goats (Fig. 6). The Spearman's rank correlation coefficient between PCV value and FAMACHA^©^ score category is very low (−0.014; *P* < 0.001) but very significant, due to the large sample size, with an indication of a negative correlation, as expected. Importantly, however, only on nine occasions were animals that had not been identified as being anaemic on FAMACHA^©^ scoring found to have haematocrits less than 20% on subsequent PCV determination. While accurate data for loss of goats from the herds (whether due to mortality, sale, theft or other reason) could not be obtained in the present study, it seems reasonable to speculate that the examination of the goats for anaemia would be a useful tool for the farmers to use. The FAMACHA^©^ system was not designed to be used on its own as the only method of control for haemonchosis on a farm. Obviously, farmers should continue to be vigilant for other indicators of ill health such as lethargy and poor body condition.

The present study shows that symptomatic anthelmintic treatment in combination with mid-summer tactical anthelmintic treatment appear to be useful strategies for the control of *H. contortus* in indigenous goats raised extensively on communal pastures in KwaZulu-Natal Province, South Africa. The use of COWP shows excellent potential as a tactical anthelmintic treatment in this and other similar agro-ecological zones.

## Figures and Tables

**Table 1a:** The analysis of variance table (ANOVA) for pre- and three periods post-COWP administration for log_10_ faecal egg counts.

Source	22 January 2008 Pre-COWP			5 February 2008 2 weeks post-COWP			4 March 2008 6 weeks post-COWP			1 April 2008 10 weeks post-COWP		
	d.f.^b^	m.s.^c^	F pr.^d^	d.f.	m.s.	F pr.	d.f.	m.s.	F pr.	d.f.	m.s.	F pr.
Area	2	0.186	0.819	2	6.476	0.004	2	0.178	0.650	2	4.126	0.016
Treat^a^	1	1.013	0.298	1	35.335	<.001	1	0.030	0.787	1	1.266	0.256
Age	1	0.079	0.771	1	1.534	0.251	1	4.016	0.002	1	7.088	0.008
Area × Treat	2	0.300	0.728	2	0.572	0.610	2	0.180	0.646	2	0.753	0.464
Area × Age	2	0.029	0.969	2	2.202	0.152	2	0.032	0.925	2	0.654	0.513
Treat × Age	1	0.063	0.794	1	0.667	0.448	1	0.141	0.559	1	0.843	0.354
Area × Treat × Age	2	0.257	0.759	2	0.058	0.951	2	0.098	0.789	2	0.658	0.511
Residual	154	0.928		156	1.154		155	0.411		160	0.975	

Total	165	0.882		167	1.141		156	0.415		171	1.038	

Levene's test	*P* = 0.576			*P* = 0.025			*P* = 0.946			*P* = 0.579		

a Treatment.

b d.f. = degrees of freedom.

c m.s. = mean square.

d F pr. = Type I probability for variance ratio.

**Table 1b:** The analysis of variance table (ANOVA) for pre- and three periods post-COWP administration for packed cell volume (PCV) in percentage.

Source	22 January 2008 Pre-COWP			5 February 2008 2 weeks post-COWP			4 March 2008 6 weeks post-COWP			1 April 2008 10 weeks post-COWP		
	d.f.^b^	m.s. c	F pr.^d^	d.f.	m.s.	F pr.	d.f.	m.s.	F pr.	d.f.	m.s.	F pr.
Area	2	8.25	0.519	2	93.00	<.001	2	0.08	0.995	2	15.69	0.278
Treat^a^	1	29.86	0.124	1	184.89	<.001	1	70.53	0.028	1	15.97	0.254
Age	1	23.32	0.174	1	0.42	0.843	1	9.62	0.414	1	14.86	0.271
Area × Treat	2	16.00	0.281	2	0.39	0.964	2	10.20	0.493	2	17.19	0.247
Area × Age	2	25.97	0.129	2	24.50	0.106	2	29.55	0.131	2	7.05	0.562
Treat × Age	1	4.91	0.532	1	3.97	0.544	1	6.91	0.489	1	0.73	0.807
Area × Treat × Age	2	12.08	0.383	2	29.76	0.066	2	23.12	0.203	2	20.91	0.183
Residual	159	12.52		160	10.76		160	14.35		160	12.17	

Total	170	12.78		171	12.90		171	14.68		171	12.29	

Levene's test	*P* = 0.599			*P* = 0.716			*P* = 0.389			*P* = 0.591		

a Treatment.

b d.f. = degrees of freedom.

c m.s. = mean square.

d F pr. = Type I probability for variance ratio.

**Table 2a:** Main effect means for pre- and three periods post-COWP administration for log_10_ faecal egg counts, original mean count and number of animals sampled (*n*).

Source effect	Levels	22 January 2008 Pre-COWP	5 February 2008 2 weeks post-COWP	4 March 2008 6 weeks post-COWP	1 April 2008 10 weeks post-COWP
Area (Bergville)	Dukuza	2.793 (2020)	2.552a (1441)	3.006 (2177)	2.559a (1567)
		*n* = 70	*n* = 71	*n* = 71	*n* = 72
	Hoffenthal	2.876 (1911)	2.004b (913)	3.011 (1798)	2.499ab (936)
		*n* = 53	*n* = 53	*n* = 51	*n* = 55
	Ogade	2.949 (2202)	1.988b (1549)	2.950 (2000)	2.097b (486)
		*n* = 43	*n* = 44	*n* = 45	*n* = 45

Treatment	Control	2.768 (2158)	2.716a (2382)	2.960 (2247)	2.487 (1346)
		*n* = 82	*n* = 84	*n* = 83	*n* = 85
	COWP	2.949 (1910)	1.747b (223)	3.024 (1783)	2.351 (825)
		*n* = 84	*n* = 84	*n* = 84	*n* = 87

Age	Young	2.956(1857)	1.883 (1509)	3.354a (2958)	2.880a (2474)
		*n* = 27	*n* = 27	*n* = 27	*n* = 28
	Adult	2.841 (2067)	2.298 (1263)	2.922b (1831)	2.329b (812)
		*n* = 139	*n* = 141	*n* = 140	*n* = 144

Means within the same source effect with the same letter or letters do not differ significantly at the 5% significance level. No letters means no significant difference.

**Table 2b:** Main effect means for pre- and three periods post-COWP administration for PCV content and number of animals sampled (*n*).

Source effect	Levels	22 January 2008 Pre-COWP	5 February 2008 2 weeks post-COWP	4 March 2008 6 weeks post-COWP	1 April 2008 10 weeks post-COWP
Area (Bergville)	Dukuza	25.26	24.96a	25.45	26.1
		*n* = 72	*n* = 72	*n* = 72	*n* = 72
	Hoffenthal	25.98	27.28b	25.41	26.96
		*n* = 55	*n* = 55	*n* = 55	*n* = 55
	Ogade	25.18	25.25a	24.8	26.35
		*n* = 44	*n* = 45	*n* = 45	*n* = 45

Treatment	Control	25.14	24.82b	24.78b	26.24
		*n* = 84	*n* = 85	*n* = 85	*n* = 85
	COWP	25.79	26.72a	25.74a	26.64
		*n* = 87	*n* = 87	*n* = 87	*n* = 87

Age	Young	25.63	25.38	23.93	25.35
		*n* = 28	*n* = 28	*n* = 28	*n* = 28
	Adult	25.44	25.86	25.52	26.66
		*n* = 143	*n* = 144	*n* = 144	*n* = 144

Means within the same source effect with the same letter or letters do not differ significantly at the 5% significance level. No letters means no significant difference.

**Table 3:** The calculated percentage efficacy, mean FECs (range) pre- and post-COWP administration and number of animals sampled (*n*) for the control (C) and treated (COWP) groups of goats per area and age group.

Area	Goat age group	Mean FEC pre-COWP		Mean FEC post-COWP		Calculated % efficacy
		C	COWP	C	COWP	
All	All	2652	2347	2709	264	89
		(200–11533)	(200–13300)	(0–14433)	(0–1800)	
		*n* = 66	*n* = 73	*n* = 66	*n* = 73	

All	Young	2572	1591	2736	246	85.5
		(200–8433)	(237–5133)	(0–9167)	(0–867)	
		*n* = 12	*n* = 11	*n* = 12	*n* = 11	

All	Adults	2669	2482	2703	267	89.4
		(267–11533)	(200–13300)	(0–14433)	(0–1800)	
		*n* = 54	*n* = 62	*n* = 54	*n* = 62	

Hoffenthal	All	2512	2114	2221	165	91.2
		(267–10833)	(200–2233)	(0–14433)	(0–567)	
		*n* = 19	*n* = 24	*n* = 19	*n* = 24	

Hoffenthal	Young	1478	1093	1689	200	84
		(367–3467)	(267–2233)	(33–4867)	(0–567)	
		*n* = 3	*n* = 5	*n* = 3	*n* = 5	

Hoffenthal	Adults	2706	2383	2321	156	92.4
		(267–10833)	(200–533)	(0–14433)	(0–100)	
		*n* = 16	*n* = 19	*n* = 16	*n* = 19	

Ogade	All	2737	2419	3489	130	95.8
		(200–6667)	(567–4267)	(0–7133)	(0–167)	
		*n* = 18	*n* = 19	*n* = 18	*n* = 19	

Ogade	Young	3878	4267	1756	0	100
		(200–6467)	-4267	(0–5267)	0	
		*n* = 3	*n* = 1	*n* = 3	*n* = 1	

Ogade	Adults	2509	2317	3836	137	96.1
		(633–6667)	(567–1867)	(0–7133)	(100–167)	
		*n* = 15	*n* = 18	*n* = 15	*n* = 18	

Dukuza	All	2690	2489	2545	428	81.8
		(333–11533	(237–13300)	(0–11667)	(0–1800)	
		*n* = 29	*n* = 30	*n* = 29	*n* = 30	

Dukuza	Young	2467	1553	3750	340	85.6
		(600–8433)	(237–5133)	(167–9167)	(0–867)	
		*n* = 6	*n* = 5	*n* = 6	*n* = 5	

Dukuza	Adults	2748	2676	2230	445	79.5
		(333–11533)	(1967–13300)	(0–11667)	(167–1800)	
		*n* = 23	*n* = 25	*n* = 23	*n* = 25	

**Table 4:** Numbers of goats assigned a FAMACHA^©^ score of 3, 4 or 5 and treated with 12 mg/kg levamisole at each visit for the control group and the COWP-treated group. (Total number of goats scored in each group in parenthesis.) COWP treatment was during the visit of 22 January 2008.

Date	16 October 2007	13 November 2007	11 December 2007	08 January 2008	22 January 2008	05 February 2008	04 March 2008	01 April 2008	29 April 2008	27 May 2008	24 June 2008	22 July 2008	19 August 2008	16 September 2008	14 October 2008
Control	66 (85)	45 (85)	39 (85)	21 (85)	41 (84)	42 (85)	42 (85)	42 (85)	26 (82)	47 (85)	32 (85)	24 (84)	15 (84)	10 (85)	21 (84)
COWP	68 (87)	55 (87)	40 (87)	28 (87)	46 (87)	38 (87)	48 (87)	41 (87)	41 (84)	51 (87)	32 (87)	28 (87)	16 (86)	17 (84)	31 (87)
